# Experimental Verification of Three-Degree-of-Freedom Electromagnetic Actuator for Image Stabilization

**DOI:** 10.3390/s20092485

**Published:** 2020-04-27

**Authors:** Akira Heya, Katsuhiro Hirata

**Affiliations:** Department of Adaptive Machine Systems, Osaka University, 2-1 Yamadaoka, Suita, Osaka 565-0871, Japan; k-hirata@ams.eng.osaka-u.ac.jp

**Keywords:** three-degree-of-freedom actuator, electromagnetic actuator, image stabilization, multiple-degree-of-freedom mechanism

## Abstract

Image deteriorations due to vibrations have become a problem in autonomous systems such as unmanned aerial vehicles, robots, and autonomous cars. To suppress the vibration, a camera stabilizer using a gimbal mechanism is widely used. However, the size and weight of the system increase because the conventional image stabilization systems require some actuators and links to drive in multi-axes. In order to solve these problems, we proposed a novel three-degree-of-freedom (3DOF) electromagnetic actuator for image stabilization. The actuator can be driven by only three-phase and has a simple structure and control system. This paper describes the experimental verification of the proposed actuator. The torque characteristics are clarified, and the analysis and measured torque characteristics are compared to verify the analysis validity. For verifying the dynamic performance, the frequency characteristics are measured. The effectiveness of the proposed magnetic structure and operating principle are investigated.

## 1. Introduction

Environment recognition is an important task for autonomous mobile systems such as unmanned aerial vehicles (UAVs), walking robots, and autonomous cars. These systems are necessary to collect image information using a camera during moving, and a high space recognition is required in an actual environment. In contrast, the decrease of the recognition accuracy due to vibrations under operation has become problems [1,2,3].

We focused on the relationship between an eyeball and a camera system. A human eye obtains a wide-angle and high-quality vision by rotating the eyeball with a retina and a crystalline lens using six muscles. The retina and crystalline lens correspond to an imaging device and a lens of a camera, respectively. Therefore, the camera mechanism with the rotating unit which consists of the imaging device and lens is suitable for the robotic eye system. Rotational vibrations in the actual environment have three-degree-of-freedom (3DOF) motion. To suppress the image deterioration from the multi-DOF vibration, a camera using a gimbal mechanism is valid [4,5,6]. It corrects an image by reverse-phase motion against the vibration. The image stabilizer using a gimbal mechanism is widely used and applied to the UAVs (e.g., Phantom 4, DJI Corp., Shenzhen, China) and video cameras for consumers (e.g., CRANE 3S, Zhiyun Corp., Guilin, China). However, these systems consist of some rotary motors and links to rotate in 3DOF. Therefore, the size and weight increase. A 3DOF actuator is expected to become a solution to these problems because it can generate 3DOF motion by only one device without links. Then, a camera system using the 3DOF actuator can be expected to have a small image stabilization system compared with conventional systems.

Various type 3DOF actuators have been proposed and developed. Multi-DOF piezoelectric [7,8] and ultrasonic actuators [9,10,11,12] are driven using a mechanical contact. They have a high holding force and a simple structure. On the other hand, the abrasion loss is large, and the high-speed rotation is difficult. The 3DOF electromagnetic actuators (3DOFEAs) can generate 3DOF motion without friction [13,14,15,16,17,18,19,20,21]. They have high responsiveness and controllability by the direct drive. However, conventional 3DOFEAs have a complicated structure and are driven by a large control device for generating the high-torque to apply a robot wrist, machine tool, and so on. In order to solve these problems, we proposed a novel 3DOFEA for image stabilization (3DOFEA-IS). The proposed actuator can generate the 3DOF motion by only three-phase and has a simple control device and structure. It has a small number of components from the proposed magnetic circuit and operating principle. The torque characteristics have been investigated by a magnetic field analysis using a three-dimensional finite element method (3-D FEM) [22]. The dynamic modeling was proposed, and its validity was verified by the comparison with the dynamic analysis using 3-D FEM, and the control performance was verified only by the numerical calculation [23]. However, experimental verification has not been reported. The validity of magnetic field analysis using 3-D FEM is not verified by an experiment. Moreover, the dynamic performance, such as frequency characteristics, is not also verified by an experiment. A prototype of the 3DOFEA-IS is not manufactured, and its detailed design is not shown. Then, the measurement of the static torque and frequency characteristics using a manufactured prototype are required.

This paper describes the experimental verification of the 3DOFEA-IS. The detailed design of the prototype is described. The torque characteristics are investigated, and the analysis and measured torque characteristics are compared to verify the analysis validity. The frequency characteristics are measured for verifying the dynamic performance. It is clarified that the 3DOFEA-IS can be driven by the proposed magnetic structure and the operating principle.

## 2. Three-Degree-of-Freedom Electromagnetic Actuator for Image Stabilization

The basic structure of the mover and stator is described. The magnetic flux path generated by permanent magnets is shown. The operating principle is described.

### 2.1. Basic Structure and Magnetic Circuit

**Figure 1:**
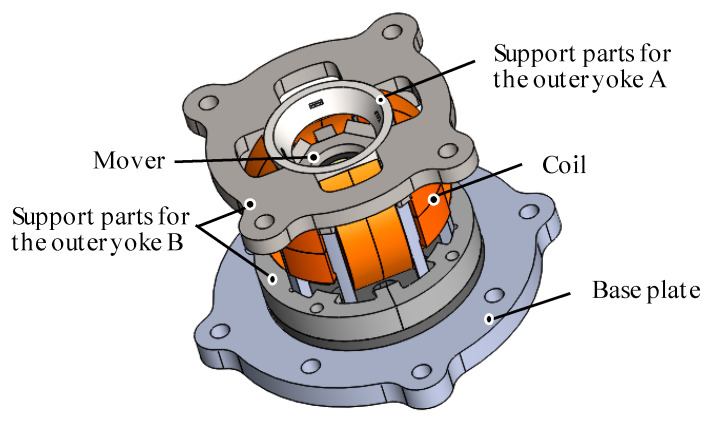
Overview of the three-degree-of-freedom electromagnetic actuator for image stabilization.

**Figure 2:**
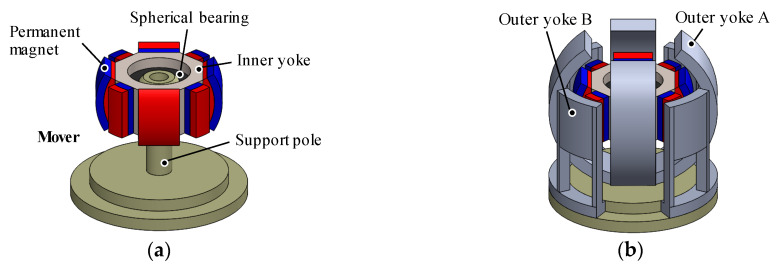
Basic structure. (**a**) Mover and the support parts; (**b**) mover and the stator without the coils.

The 3DOFEA-IS is composed of the outer stator and the inner mover (see Figure 1). The outer stator consists of coils, the outer Yoke A, and the outer Yoke B, as shown in Figure 2. The inner mover consists of eight permanent magnets and the inner yoke. The permanent magnets are arranged by every 45 deg. The coils for rotating in the *X*-axis and *Y*-axis are wound around the outer Yoke A. The coil for rotating around the *Z*-axis is wound around the outer Yoke B.

**Figure 3:**
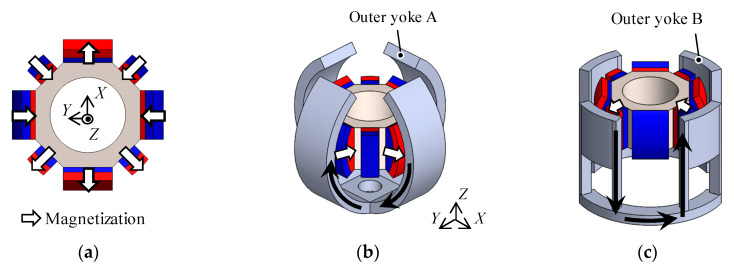
Magnetic flux path. (**a**) Mover; (**b**) magnetic flux path of the outer Yoke A and the mover; (**c**) magnetic flux path of the outer Yoke B and the mover.

The magnetization direction of the permanent magnets are shown in Figure 3a. The magnetic flux generated by the permanent magnets passes through the outer Yoke A and the inner yoke (Figure 3b), and also passes the outer Yoke B (Figure 3c). From this magnetic structure, the magnetic spring characteristics for attracting to the origin are generated. It is caused by changing the facing area between the permanent magnets and yokes when the mover rotates.

### 2.2. Operating Principle

**Figure 4:**
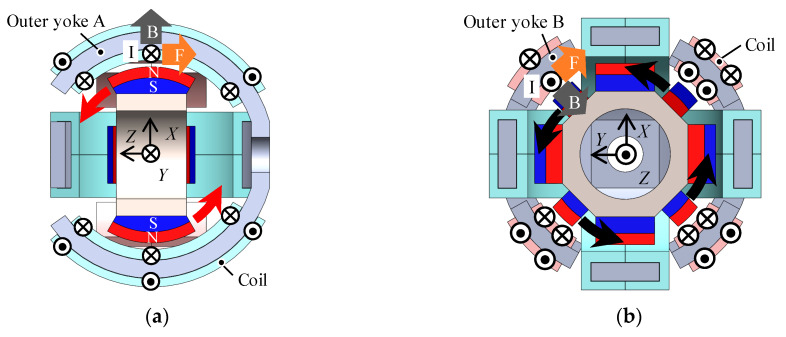
Operating principle. (**a**) Rotation in the *X*/*Y*-axis; (**b**) Rotation in the *Z*-axis.

The operating principle is based on a voice coil motor, as shown in Figure 4. A torque in the *X*-axis is generated by the Lorentz force. The coils carrying currents generate tangential direction forces that generate torque, and the mover rotates in the *X*-axis (Figure 4a). Similarly, the rotation torque in the *Y*-axis and *Z*-axis is generated and the mover rotates in the *Y*-axis (Figure 4a) and *Z*-axis (Figure 4b). The 3DOF motion is achieved by adding the torques generated by each coil. Therefore, the actuator can be independently driven by only three-phase coils.

## 3. Prototype

This section describes the design and specification of the prototype. The system configuration of the operating verification is shown. The feasibility of the operating principle is verified using the prototype.

### 3.1. Design

**Figure 5:**
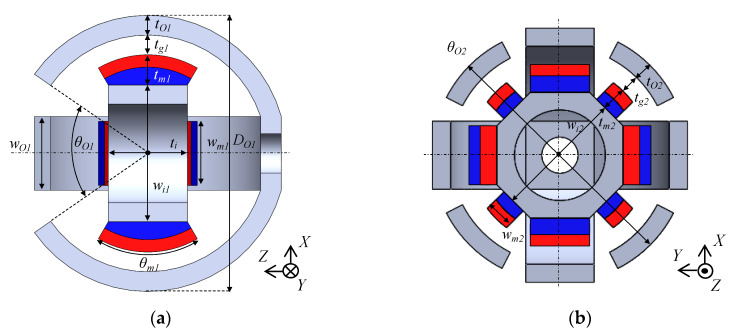
Design parameters of the actuator without the coils. (**a**) *X*–*Z* cross section; (**b**) *X*–*Y* cross section.

**Figure 6:**
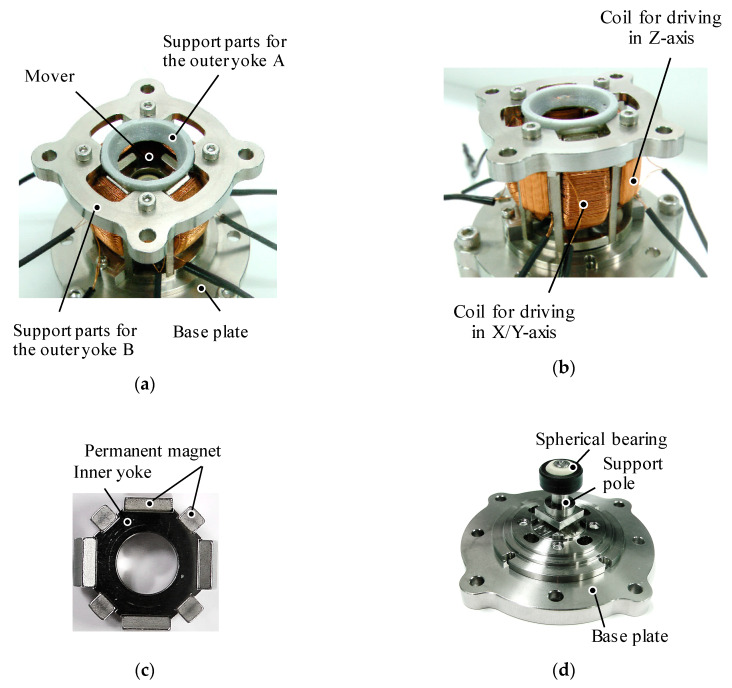
Overview of the prototype. (**a**) Top view; (**b**) Side view; (**c**) Mover; (**d**) Supporting mechanism without the stator.

**Figure 7:**
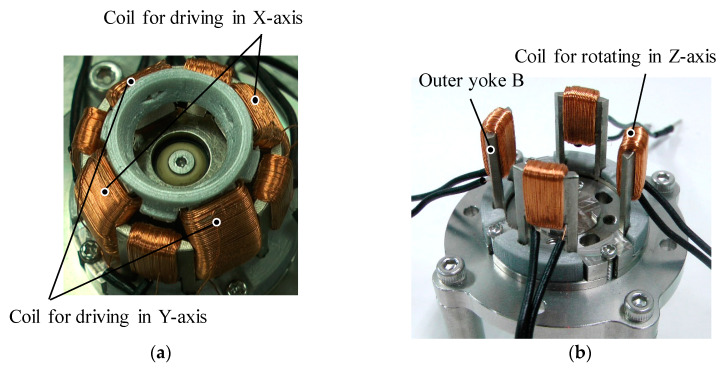
Coil structure. (**a**) Whole view without the support parts for the outer Yoke B; (**b**) Outer Yoke B and the coil for rotating in the *Z*-axis.

The design parameters of the prototype are shown in Figure 5. Its values are listed in Table 1. The dimensions of the yokes and permanent magnets are designed through a magnetic field analysis using a 3-D FEM. As a design requirement, the outer diameter of the actuator is limited to 30 mm. The dimensions are determined by trial and error; an optimization method was not used. The overview of the prototype is shown in Figure 6. The coil structures are shown in Figure 7. The mover is supported by a spherical bearing (KGLM-03, igus Corp., Cologne, Germany) which is made of a non-magnetic material. It is located at the center of the mover by the supporting pole. The outer Yoke A is fixed by the supporting parts A made of a non-magnetic material. The outer Yoke B is constructed by separated parts. The movable angle in the *X*-axis, the *Y*-axis is ±25 deg., and that in the *Z*-axis is ±5 deg.

### 3.2. Operating Verification

**Figure 8:**
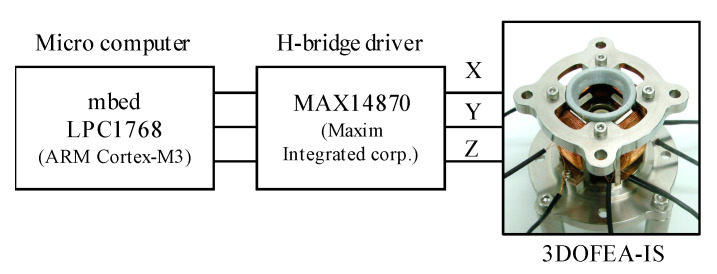
System configuration for the operating verification.

To verify the operation, the currents are applied to the coils using H-bridge circuits (MAX14870, Maxim Integrated Corp., San Jose, California, CA, USA), as shown in Figure 8. The input voltage is 12 V. The coils are excited under a PWM control. The PWM frequency is 20 kHz. The input signal is processed using a micro-computer (mbed LPC1768, ARM Cortex-M3, NXP Semiconductors N. V., Eindhoven, The Netherlands).

**Figure 9:**
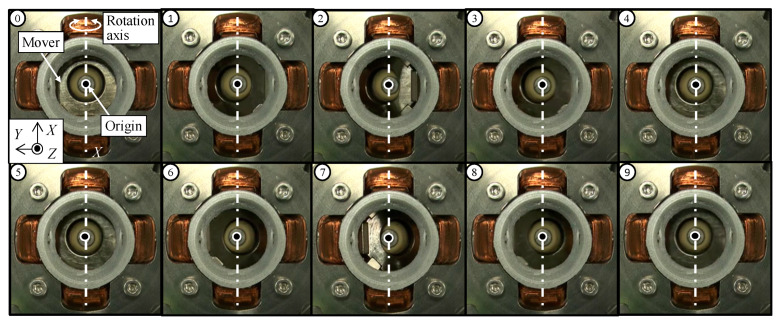
Operating verification of the single-axis drive (*X*-axis).

**Figure 10:**
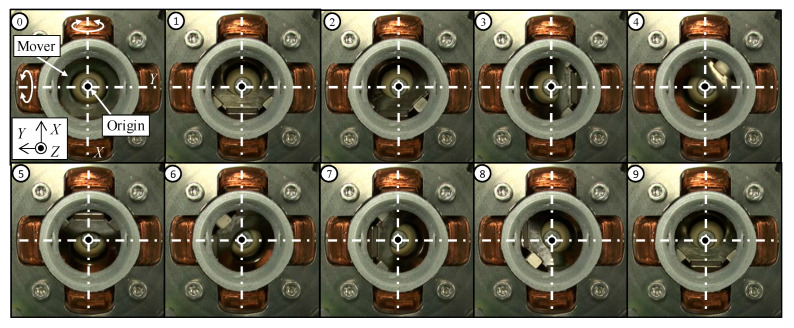
Operating verification of the multi-axes drive.

The sequential views when driving in the single-axis are shown in Figure 9. The coil for rotating in the *X*-axis is only excited. From the sequential views, it is found that the slope in the upper surface of the mover is changed around the *X*-axis. It shows that the mover is rotated in the *X*-axis. Next, to verify the multi-DOF motion, the coils for rotating in the *X*-axis and *Y*-axis are excited simultaneously. The sequential views when driving in multi-axes are shown in Figure 10. From Figure 10, the slope in the upper surface of the mover is changed, they show that the mover rotates in a circular motion. From these experimental results, it is shown that the mover can be driven by exciting each coil.

## 4. Torque Characteristics

This section describes the torque characteristics of the 3DOFEA-IS. First, the analysis conditions of a magnetic field analysis using a 3-D FEM are described, and the calculated torque characteristics are shown. Second, the experimental setup and measured torque characteristics are shown. The analysis and measured results are compared, and the analysis validity is discussed.

### 4.1. Analysis Conditions

**Figure 11:**
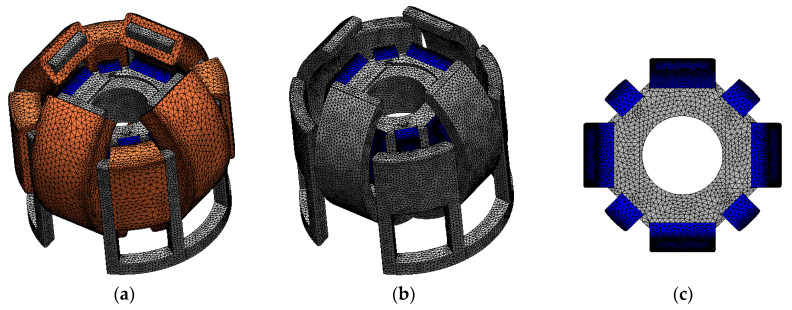
Three-dimensional (3-D) mesh model without the air region (**a**) Whole view; (**b**) Mover and stator without the coils; (**c**) Mover.

**Figure 12:**
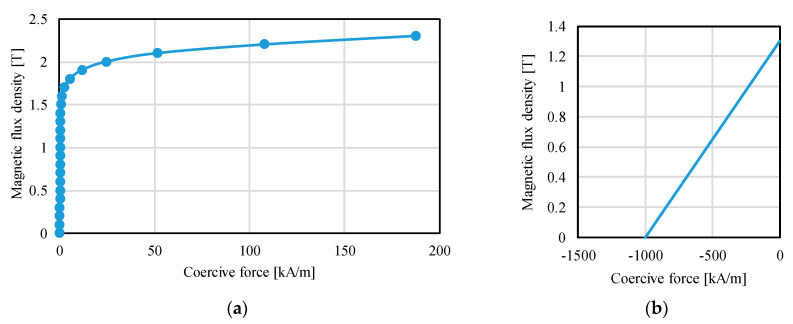
Magnetic characteristics of the magnetic material. (**a**) Yoke; (**b**) Permanent magnet.

The T-Ω method was employed to compute the static torque [24,25]. A commercial software (MagNet, Infolytica Corp., Montreal, QC, Canada) was used for magnetic field analysis. The 3-D mesh model without the air region is shown in Figure 11. The analysis conditions are listed in Table 2. The mesh model of the actuator component and air region are finely divided to reduce the variation of the calculated torque in trial and error. The calculated torque using the determined mesh model was almost the same compared with the calculated torque using the more finely divided mesh model. The yokes are made of an electromagnetic soft iron; its BH curve is shown in Figure 12a. The magnetic characteristics of the permanent magnet are calculated as linear characteristics, as shown in Figure 12b. The residual magnetic flux density of the permanent magnets is 1.3 T (N42H), and the coercive force is 1.0 × 10^6^ A/m. In this paper, the detent torque is defined as the torque when the coils are not excited. The current torque is defined as the difference of the output torque and detent torque. The torque constant is calculated as the current torque per current.

### 4.2. Analysis Results and Discussion

**Figure 13:**
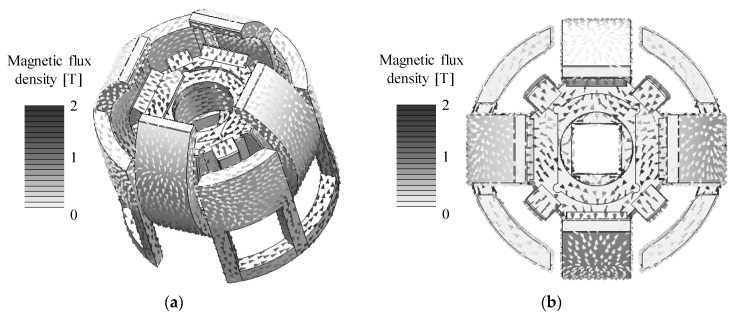
Calculated magnetic flux density distribution. (**a**) Whole view; (**b**) Top view.

**Figure 14:**
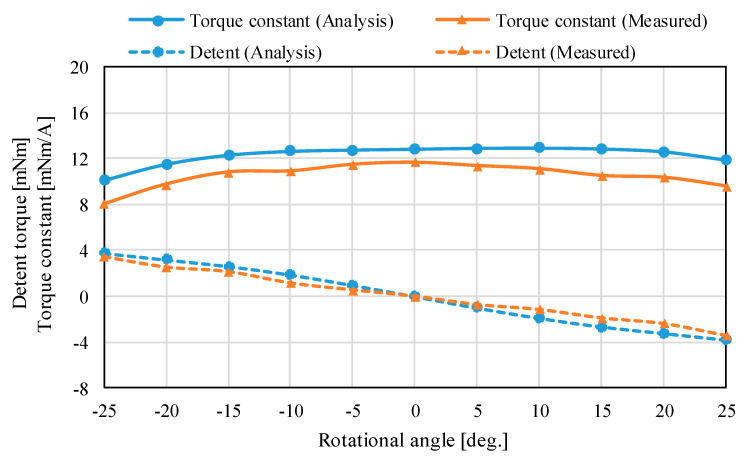
Torque characteristics in the *X*-axis.

**Figure 15:**
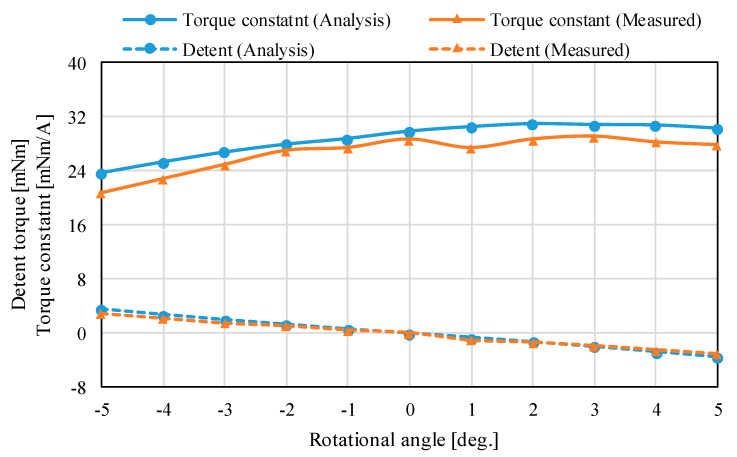
Torque characteristics in the *Z*-axis.

The magnetic flux density distributions are shown in Figure 13. The magnetic fluxes generated by the permanent magnets pass through the outer Yokes A and B. The analysis results of the torque characteristics in the *X*-axis and *Z*-axis are shown in Figure 14 and Figure 15. The detent torque characteristics in the *X*-axis and *Z*-axis have the negative slope and origin symmetric in the rotation, respectively. It shows that the detent torque works as the magnetic spring. Therefore, a mechanical spring is not required for returning to the origin position. The torque constants in the *X*-axis and *Z*-axis are positive values in the movable range. The maximum torque is generated at the origin position. The torque characteristics have asymmetric characteristics in the rotation. It is caused by the cancellation of the bias magnetic flux when the coils are excited.

### 4.3. Experimental Setup

**Figure 16:**
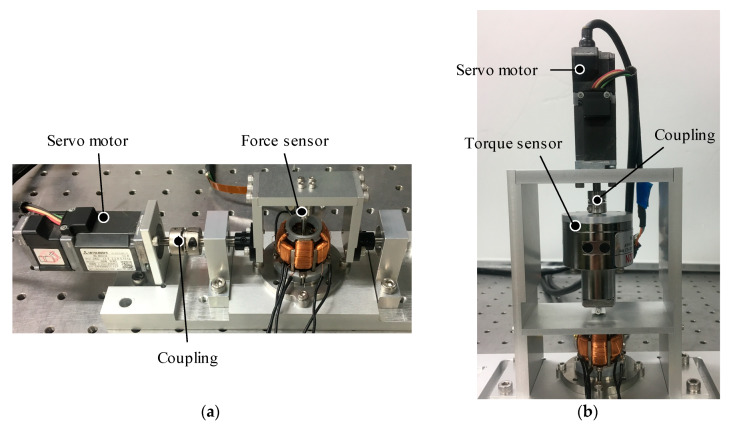
Experimental setup of the torque measurement. (**a**) Rotation in the *X*-axis; (**b**) Rotation in the *Z*-axis.

The experiment environments of the torque measurement are shown in Figure 16. The torque characteristics in the *X*-axis and *Z*-axis are measured using a force sensor (MAF-3, WACOH-TECH Inc., Toyama, Japan) and a torque sensor (TCF02N, Tohnichi Mfg. Co., Tokyo, Japan), respectively. The mover is forcibly rotated by a servo motor (HG-AK0336, Mitsubishi Electric Corp., Tokyo, Japan). The servo motor is controlled by a PLC (QD75MH2 and Q03UDCPU, Mitsubishi Electric Corp.) and a servo amp (MR-J4W2-0303B6, Mitsubishi Electric Corp.). The rotational shaft of the motor is connected to the mover using links and mechanical coupling. The resolution of the encoder is 262,144 pulses/rev. To measure the torque constant of the actuator, DC currents to the actuator are supplied by a bipolar amplifier (HAS4014, NF Corp., Kanagawa, Japan).

The detent torque characteristics in the *X*-axis are measured by the force sensor when the coils are not excited (see Figure 16a). The force sensor is arranged between the output shaft of the mover and rotational link. This actuator has magnetic spring characteristics in non-current, therefore, the reaction force can be measured by rotation using the servo motor. The torque constants in the *X*-axis are also measured by the force sensor. Similarly, the detent torque characteristics and torque constants in the *Z*-axis are measured using the torque sensor, as shown in Figure 16b.

### 4.4. Measured Results and Discussion

The measured torque characteristics in the *X*-axis and *Z*-axis are shown in Figure 14 and Figure 15. The measured detent torque characteristics have a negative slope and origin symmetric, therefore, these characteristics work as the magnetic spring for attracting to the origin. This feature was also found by the 3-D FEM analysis. The measured detent characteristics have small shakiness. It is caused by measurement errors from the experimental environment such as a rattling of the supporting mechanism and a small misalignment in the force sensor. The torque constant characteristics also have shakiness from the experiment error. However, the detent torque characteristics have linearity, the torque constants are positive values in the rotational range. The torque constant characteristics in the *X*-axis are almost flat; on the other hand, that in the *Z*-axis has a slope. It is caused by the cancellation of the bias magnetic flux when the coils for rotating in the *Z*-axis are excited.

The root means square errors (RMSEs) between the analysis and measured results in the *X*-axis and *Z*-axis are 0.49 mNm and 0.33 mNm, respectively. The measured torque constants are positive values in the movable range. The RMSEs between the analysis and measured results in the *X*-axis and *Z*-axis are 0.61 mNm/A and 2.06 mNm/A, respectively. The error ratios of the RMSE and maximum torque constant are 5.2% and 7.1%. From the comparison, the analysis results are roughly matched with the measured results. The errors between the analysis and experiment are caused by the mechanism error based on assembling errors of the yoke part and processing errors of the permanent magnet.

## 5. Frequency Characteristics

The frequency characteristics are measured to verify the dynamic characteristics. The angle of the mover is measured using a laser displacement sensor. The gain of the frequency characteristics is calculated as the ratio of the amplitude of rotational angle and input voltage.

### 5.1. Experimental Environment

**Figure 17:**
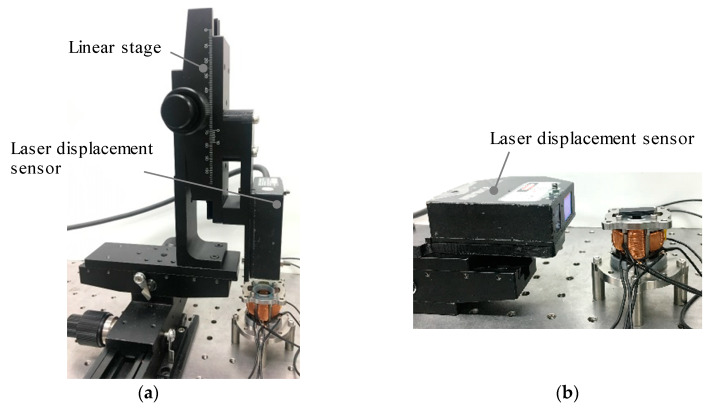
Experimental setup of the frequency characteristics. (**a**) Rotation in the *X*-axis; (**b**) Rotation in the *Z*-axis.

**Figure 18:**
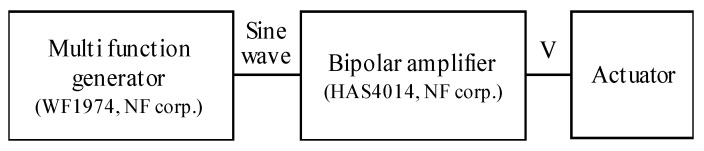
Input voltage for the frequency characteristics.

The experimental environments for measuring the frequency characteristics are shown in Figure 17. The angle is measured using a laser displacement sensor (LK-G35, KEYENCE Corp., Osaka, Japan). Displacement due to the rotation of the mover is converted to an angle in each axis. The repeat accuracy of the sensor is 0.05 μm. The gain of frequency characteristics is calculated as a ratio of the amplitude of the rotational angle and input voltage. The sine wave voltage is given by a function generator (WF1974, NF Corp., Kanagawa, Japan) and the bipolar amplifier, as shown in Figure 18.

### 5.2. Results and Discussion

**Figure 19:**
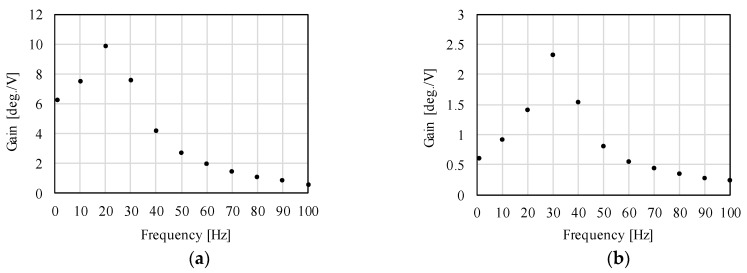
Measured results of the frequency characteristics. (**a**) Rotation in the *X*-axis; (**b**) Rotation in the *Z*-axis.

The measured results of the frequency characteristics are shown in Figure 19. It is observed that the proposed actuator can be driven until 100 Hz. The gains of the *X*-axis and *Z*-axis at 100 Hz are 0.62 deg./V and 0.23 deg./V, respectively. The resonance frequencies in the *X*-axis and *Z*-axis are around 20 Hz and 30 Hz, respectively. The gain sharply increases around the resonance frequency. By contrast, the gain becomes small and gradually decreases attendant on an increase of frequency.

In this paper, the losses from thermal heating effects caused by eddy current and friction are not measured. The thermal heating does not occur during the experiment in a short time, however, the friction in the spherical bearing is not expected to be small because the bearing is slide bearing. In long-term operation, it is better that the friction is reduced by using a spherical rolling ball bearing. Although the eddy current loss becomes large in an increase of frequency, the operating range of the actuator is not high, then, that loss is not expected to be large. In this prototype, the yokes are made of SUY, and the eddy current loss can be reduced by using a yoke made of laminated steel.

From these results, it is clarified that the proposed actuator can be driven. The gain is small in the high-frequency range; on the other hand, the target major frequency component is less than 30 Hz. The drastic increase of the gain around the resonance frequency shows that the proposed actuator can drive by low power around the resonance frequency. This paper showed only the experimental verification of the actuator in an open-loop system. When a feedback control system is designed including the actuator and controller, its operating frequency can be changed by adjusting the controller characteristics such as gain parameters of a PID controller.

## 6. Conclusions

This paper described the experimental results of a prototype of the proposed 3DOFEA-IS. The detailed design was described. The analysis and measured torque characteristics were clarified. It is shown that the proposed magnetic structure and the operating principle are valid. In addition, to verify the dynamic performance, the frequency characteristics were measured. Although the gain decreases attendant on an increase of frequency, the measured frequency characteristics show that the proposed actuator can be driven. From these experimental results, the effectiveness of the proposed actuator was clarified. The actuator can drive in 3DOF by only one device without links, thus, it is expected that the conventional image stabilization system which has three actuators and links can be downsized and simplified. In future works, we will conduct a mechanical vibration analysis, and propose a sensor-less attitude estimation for downsizing the system.

## Figures and Tables

**Table 1:** Dimensions of the actuator.

Parameter	Description	Value
*D_O_*_1_, *D_O_*_2_	Outermost radius	28.0 mm
*t_O_*_1_, *t_O_*_2_	Outer yoke thickness	2.0 mm
*t_g_*_1_	Air gap length	2.0 mm
*t_g_*_2_		2.1 mm
*t_m_*_1_	Permanent magnet thickness	3.1 mm
*t_m_*_2_		2.7 mm
*t_i_*	Inner yoke thickness	8.0 mm
*θ_m_*_1_, *θ_m_*_2_	Permanent magnet angle	60.0 deg.
*Θ_O_*_1_, *θ_O_*_2_	Outer yoke angle	70.0 deg.
*w_O_*_1_	Outer yoke width	7.5 mm
*w_O_*_2_		10.0 mm
*w_m_*_1_	Permanent magnet width	6.5 mm
*w_m_*_2_		3.0 mm
*w_i_*_1_	Inner yoke width	13.9 mm
*w_i_*_2_		14.4 mm

**Table 2:** Analysis conditions.

Number of elements	2,513,028
Number of nodes	438,221
Calculation time	14 min/step
CPU	IntelI Core(TM) i9-9900K
